# Moderating effects of the effectiveness of psychological interventions for suicide behavior and non-suicidal self-harm prevention in prison settings: a systematic review and meta-analysis

**DOI:** 10.1192/j.eurpsy.2025.285

**Published:** 2025-08-26

**Authors:** A. Pedrola-Pons, W. Ayad-Ahmed, A. De la Torre-Luque, M. Elices, E. Carballo Sánchez de Rojas

**Affiliations:** 1 Medicina Legal, Psiquiatría y Patología., Universidad Complutense de Madrid; 2 Hospital Clínico San Carlos, Madrid; 3 Instituto Hospital del Mar de Investigaciones Médicas (IMIM), Barcelona, Spain

## Abstract

**Introduction:**

Suicide remains a major cause of death in prison (Status report on prison health in the WHO European Region 2022). In comparison with adults from general population, incarcerated people are at increased risk of presenting suicide-related behaviours (Fazel S, *et al*. Lancet Psychiatry 2017; 4 946–52). Although certain studies have identified effective programs to reduce suicide in prison context (Carter A, *et al*. EClinicalMedicine 2022; 44 101-266), there is little evidence examining the relationship between moderators of effectiveness at individual and contextual levels.

**Objectives:**

This study aims to review empirical research on moderators of effectiveness of interventions in prison to reduce suicide, summarizing effect sizes across studies.

**Methods:**

For this systematic review and meta-analysis, we searched EBSCOhost, ScienceDirect, PubMed and ProQuest for articles published from 1990 to 2024. Elegible studies included those evaluating the effect of psychological interventions, delivered to adults during incarceration, on suicidal prevention. The impact of moderators covering bibliometric features (i.e. year of publication, country), methodological features of the study (i.e. sample size, mean age of participants, sex ratio, study design, assessment type and tools), suicide-related features (main outcome, previous suicide history), and other relevant variables (prison type and location, type and length of sentence) as well as psychological traits (alcohol or drugs misuse or other treatments) were also included. This review was conducted in accordance with PRISMA guidelines. Meta-analyses using random-effect models were used to pool effect sizes for moderators’ outcomes. The protocol was pre-registered with PROSPERO, CRD42024538967.

**Results:**

Of 7728 articles retrieved, 18 studies (1695 participants, 330 [19.5%] females, 756 males [44.6%], and 609 [35.9%] unknown) met the inclusion criteria. Mean ages were 32·0 years, and ethnicity data was not sufficiently reported to be aggregated. Type of prison was mostly public sector and located in rural areas. Studies were frequently conducted in UK (*n*=8; 44%) and used varying study designs; most frequently pre-post with no control group (*n*=9; 50%). On average, prevention programs in prison context were effective in decreasing suicide deaths, suicidal ideation and self-harm (*n*=14; 78%).

**Conclusions:**

Findings suggest that explanations for efficiency of psychological interventions to prevent suicide behaviour and self-harm in prison context, are moderated by physical environment, individual and psychosocial factors. Future research identifying what factors moderate treatment outcomes in suicide and self-harm prevention within prison environments could help elucidate associated factors of efficiency, helping develop potential therapeutic actions.

**Disclosure of Interest:**

None Declared

